# Supplementary material for: CRISPR GUARD protects off-target sites from Cas9 nuclease activity using short guide RNAs
Source: Nat Commun. 2020 Aug 17;11:4132. doi: 10.1038/s41467-020-17952-5 (PMC7431537; doi:10.1038/s41467-020-17952-5)
Supplement: Supplementary file 1 — Supplementary Information [file 41467_2020_17952_MOESM1_ESM.pdf]

CRISPR GUARD protects off-target sites from Cas9 nuclease activity using short guide RNAs

Coelho et al.

Supplementary Figure 1

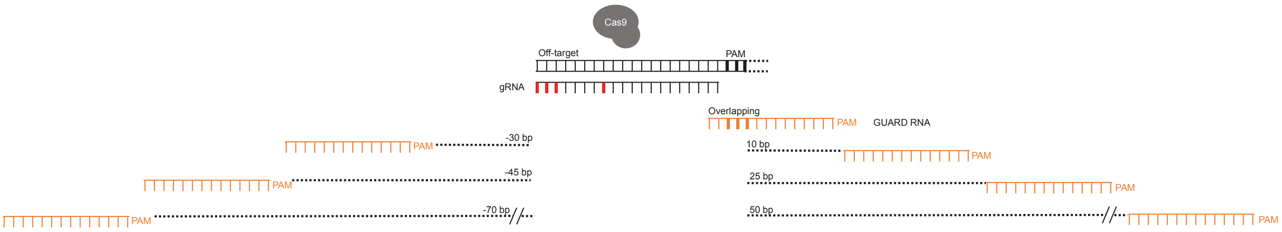

Schematic of the experiment described in Fig. 2e.

Supplementary Figure 2

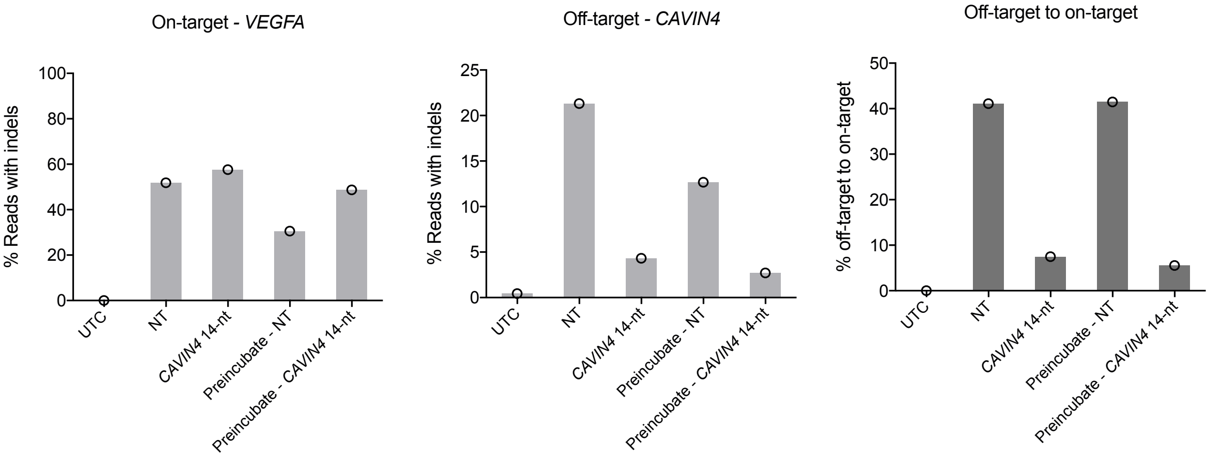

Pre-incubation of GUARD RNAs in cells does not provide additional protection of off-target sites. Indel rates from amplicon sequencing of Cas9-expressing HEK293 cells transfected with *VEGFA* on-target gRNA and *CAVIN4* proximal off-target GUARD RNA at ratio of 25:25 nM. Transfection was performed concomitantly, or by first transfecting with GUARD RNA and 18 h later, transfecting with the on-target gRNA (‘pre-incubate’ condition). The ratio of off-target to on-target is represented in the right-hand panel.

Source data are provided as a Source Data file.

Supplementary Figure 3

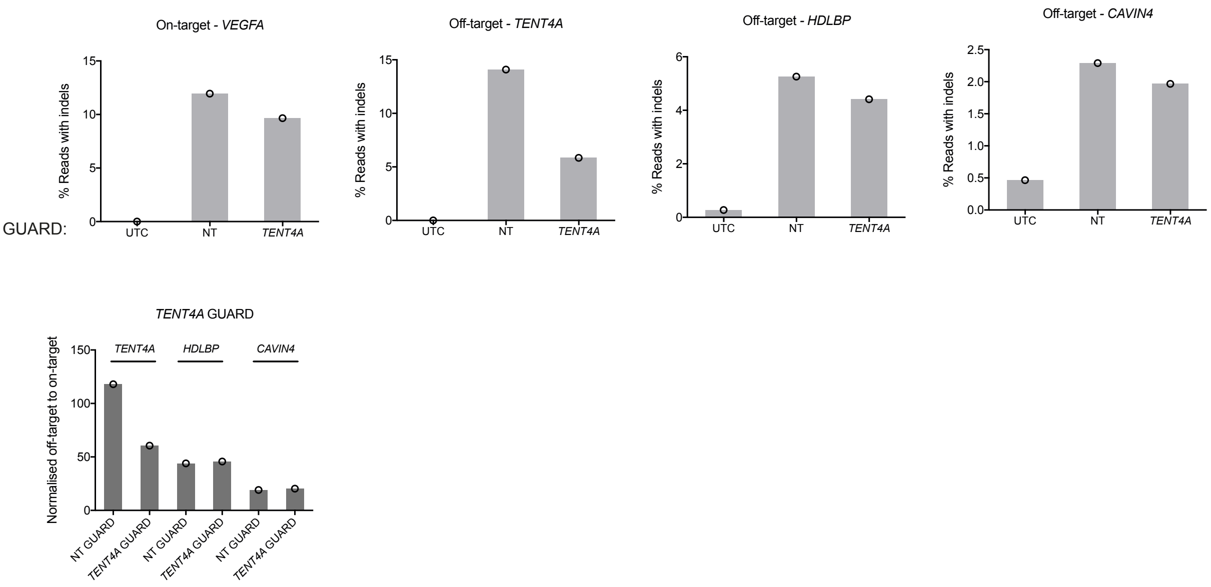

CRISPR GUARD protection of one off-target site does not lead to increased off-target mutagenesis at other sites. Amplicon sequencing analysis of HEK293 cells electroporated with wild-type Cas9 protein complexed with *VEGFA* on-target gRNA, and dCas9 protein complexed with *TENT4A* GUARD RNA. On-target cutting (*VEGFA*), and *TENT4A, HDLBP* and *CAVIN4* proximal off-target sites were analysed for indel formation. The ratio of off-target to on-target is represented in the lower panel.

Source data are provided as a Source Data file.

Supplementary Figure 4

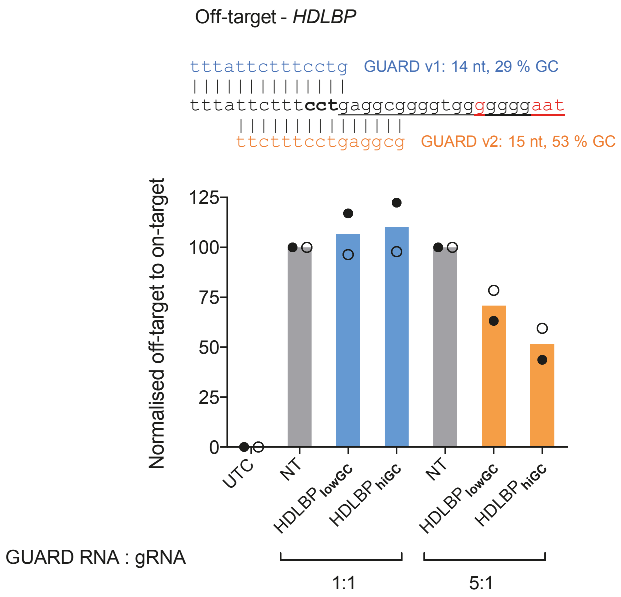

Rational redesign of poorly performing GUARD RNAs by increasing GC-content. Shown is the *VEGFA* gRNA off-target region *HDBLP*, with the mismatched gRNA and two distinct GUARD RNA designs. Indel rates for 25:25 nM and 125:25 nM GUARD RNA to gRNA ratios from NGS of amplicons from Cas9-expressing HEK293 cells are expressed as an off-target to on-target ratio. Mean of two independent experiments with symbols representing each replicate.

Source data are provided as a Source Data file.

Supplementary Figure 5

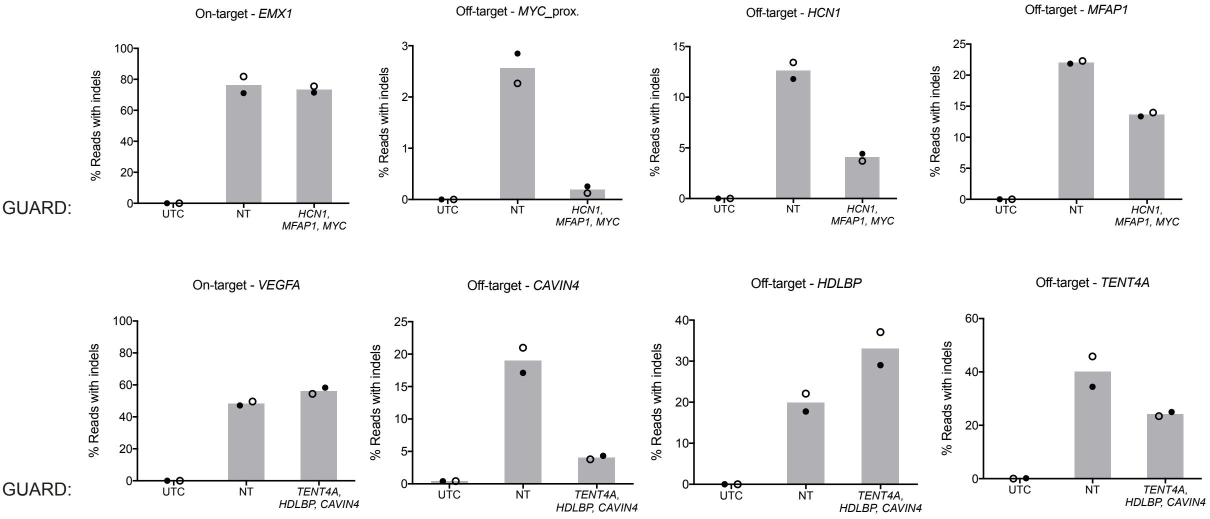

Multiplexed delivery of GUARD RNAs provides protection against multiple Cas9 off-targets simultaneously. Indel rates from amplicon sequencing of Cas9-expressing HEK293 cells transfected with *EMX1* or *VEGFA* on-target gRNAs, and three GUARD RNAs (25:25:25:25 nM ratio). Mean of two independent experiments with symbols representing each replicate.

Source data are provided as a Source Data file.

Supplementary Figure 6

a

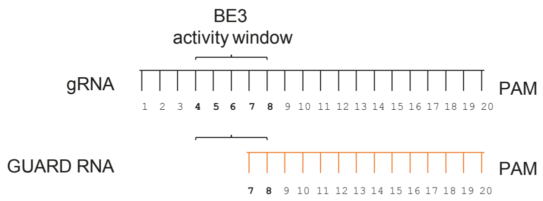

b

On-target EMX1	5′ GAGTCCGAGCAGAAGAAGAA**GGG** 3′
OFF MFAP1	5′ GAGTCTAAGCAGAAGAAGAA**GAG** 3′
GUARD1	5′ TAAGCAGAAGAAGAA**GAG** 3′
OFF MYC proximal	5′ GAGTCCTAGCAGGAGAAGAA**GAG** 3′
GUARD2	3′ **GGT**CGTACTGGACTCAG 5′

On-target VEGFA	5′ GACCCCCTCCACCCCGCCTCCGG 3′
OFF HDLBP	5′ ATTCCCCCCCACCCCGCCTC**AGG** 3′
GUARD1	3′ **GGG**GCGGAGTCCTTTCTT 5′
OFF CAVIN4	5′ ACACCCCCCCACCCCGCCTC**AGG** 3′
GUARD2	5′TCAGGACGTCCAAC**AGG** 3′

c

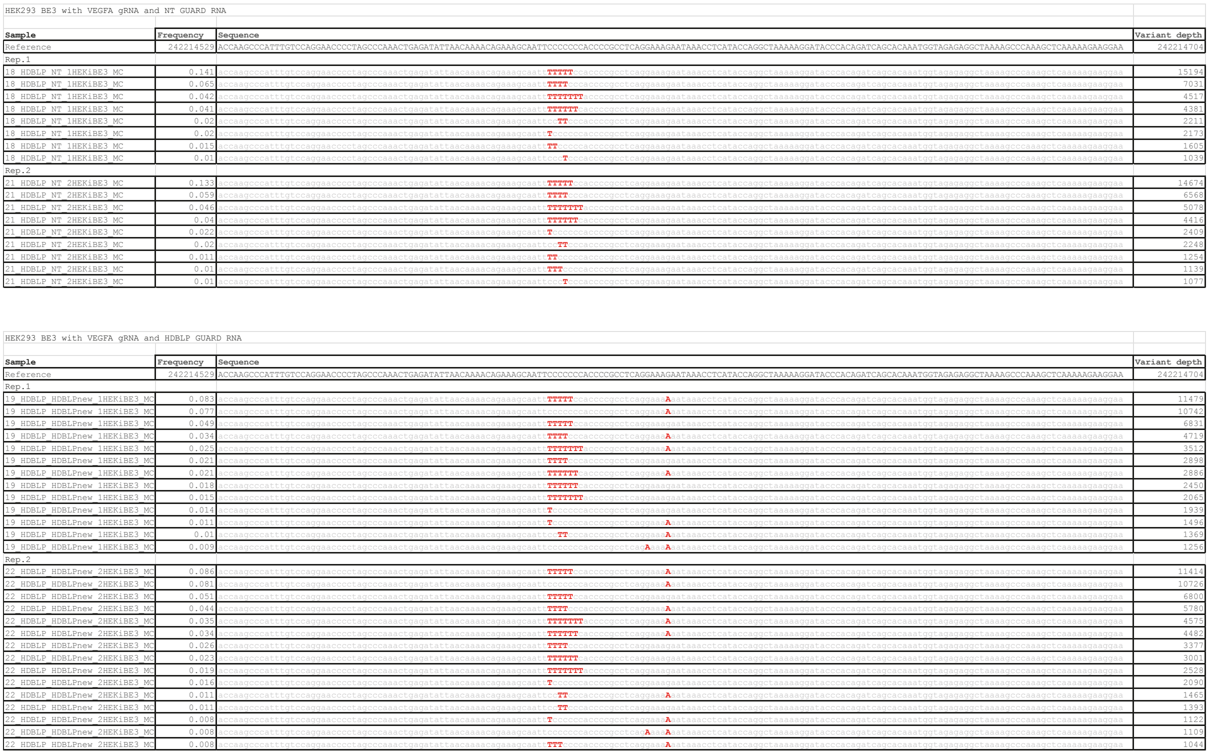

(a) Schematic representation of the predicted base editing activity window for full-length, 20-nt gRNAs and short GUARD RNAs.

(b) Sequence and position of the gRNA on-target, off-target sites and GUARD RNAs used for protection, annotated as in Figure 1.

(c) Linked and unlinked base editing events caused by on-target *VEGFA* gRNA binding and *HDLBP* GUARD RNA binding. Base editing rates from amplicon sequencing of BE3-expressing HEK293 cells transfected with *VEGFA* on-target gRNA (25 nM) and the indicated GUARD RNA (125 nM). NGS reads for the off-target region are shown with edited nucleotides highlighted in red.

Source data are provided as a Source Data file.

Supplementary Figure 7

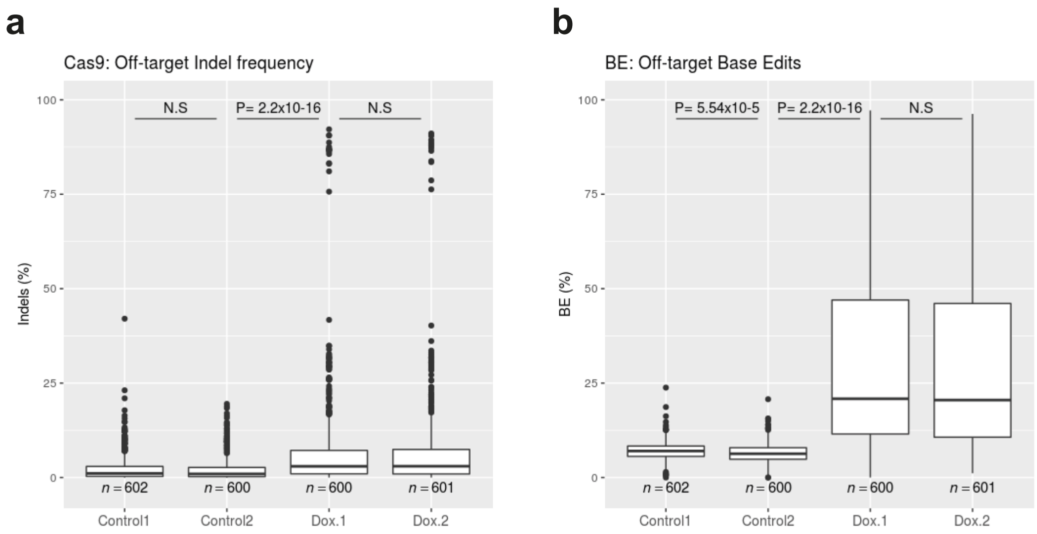

(a) Box and whiskers plot of off-target indel frequency for each replicate of the Cas9 screen.

(b) Box and whiskers plot of off-target SNP frequency for each replicate of the BE3 screen.

Center line, median; box limits, upper and lower quartiles; whiskers, 1.5x interquartile range; points, outliers. Unpaired, two-tailed student's *t*-test.

Supplementary Figure 8

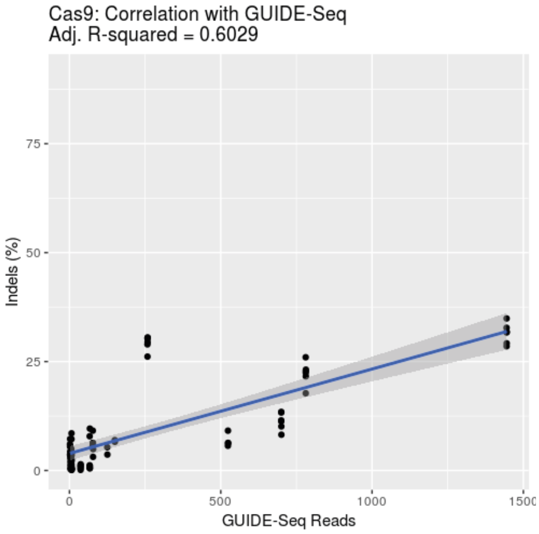

Dot plot showing the correlation of non-targeting GUARD RNA editing rates (% reads with indels) and publicly available GUIDE-Seq read counts for off-targets used in the Cas9 screen. The shaded area represents the 95 % confidence interval.

Supplementary Figure 9

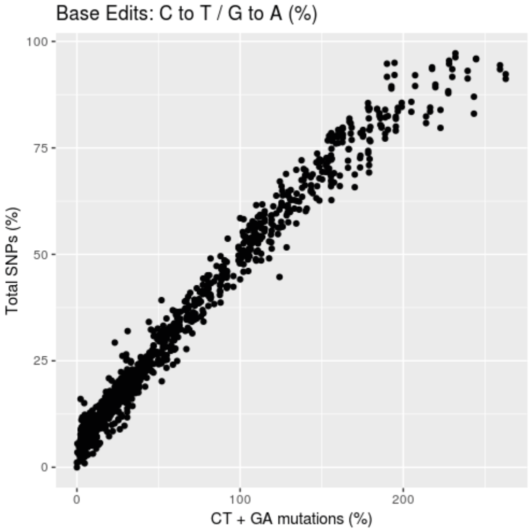

For the BE3 GUARD RNA screen, correlation between total SNPs (% of reads) and C->T and G->A (sum % of reads). As some reads contained both C->T and G->A mutations, the % exceeds 100 % in some cases. Data represent all GUARD RNAs from two independent replicates of doxycycline-treated cells.

Supplementary Figure 10

a

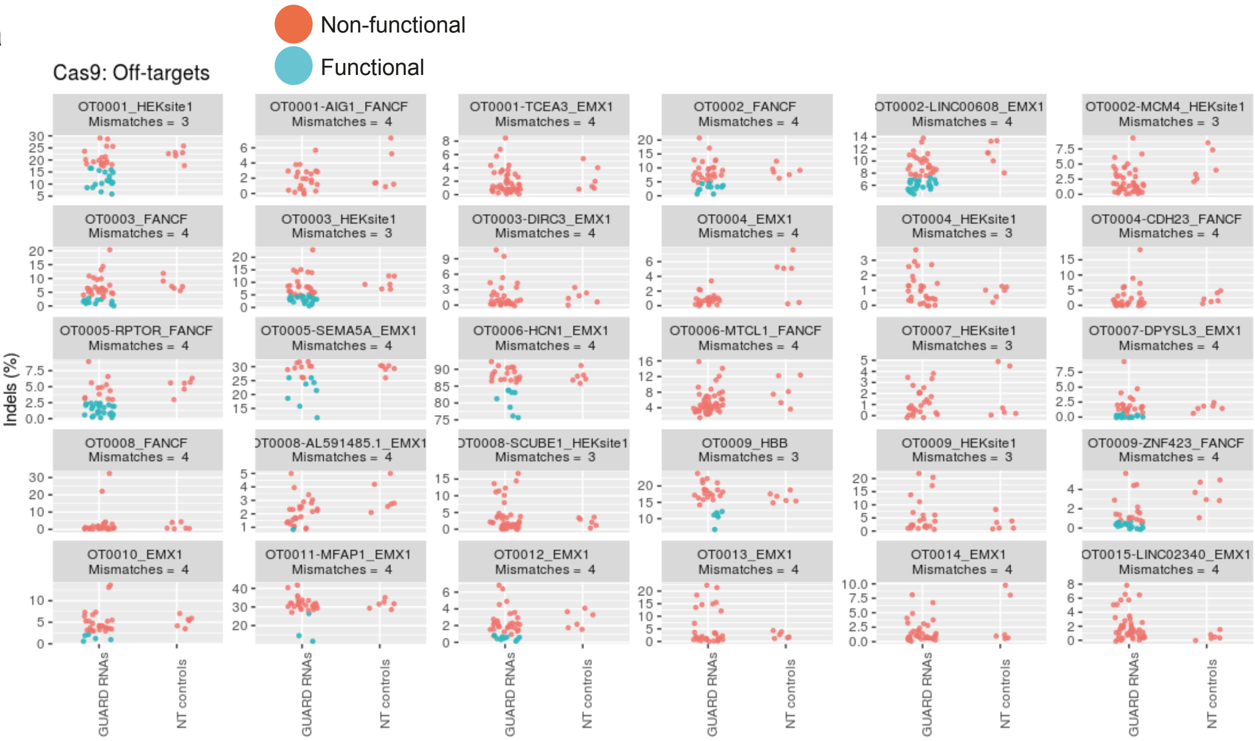

b

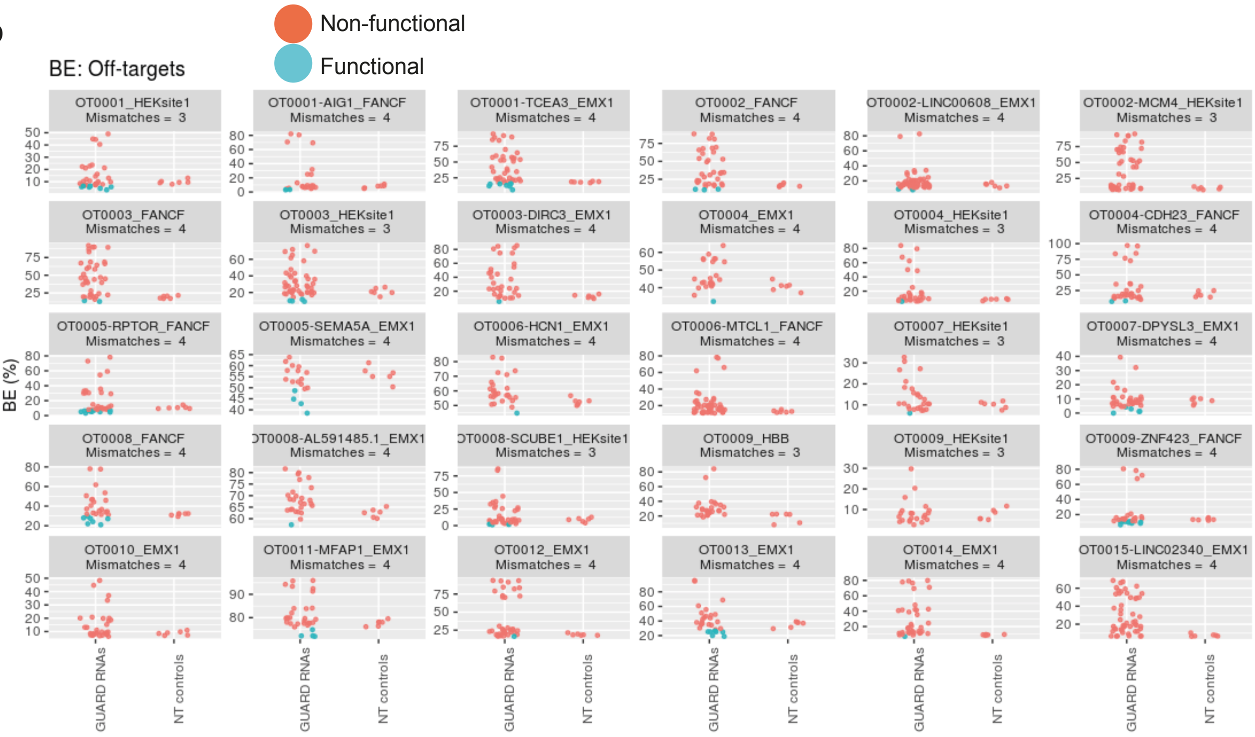

(a) GUARD RNA performance for Cas9 and,

(b) BE3. Indel rates (% of NGS reads with indels) and BE rates (% of NGS reads with SNPs) for a 30 off-target sites. Off-targets are labelled with an identifier, gene name (if genic), gRNA name, and the number of mismatches between the gRNA and the off-target site. Data are pooled from two independent experiments from cells treated with doxycycline. Each point represents a GUARD RNA and are compared to three non-targeting (NT) control RNAs. GUARD RNAs are classed as “functional” if they reduce off-target editing by at least two standard deviations from the mean of the NT controls.

Supplementary Figure 11

a

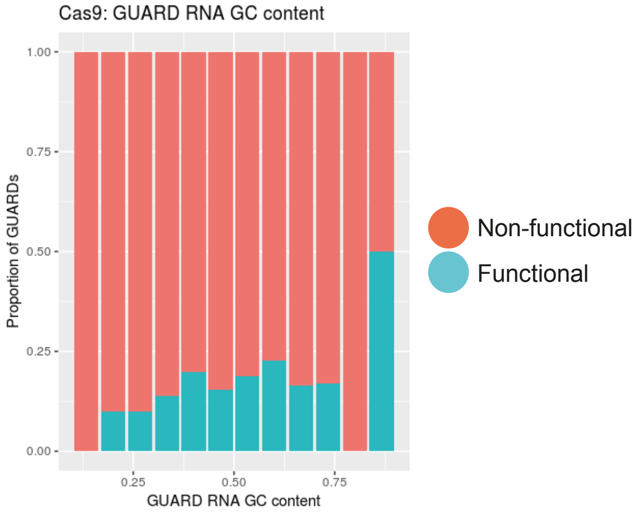

b

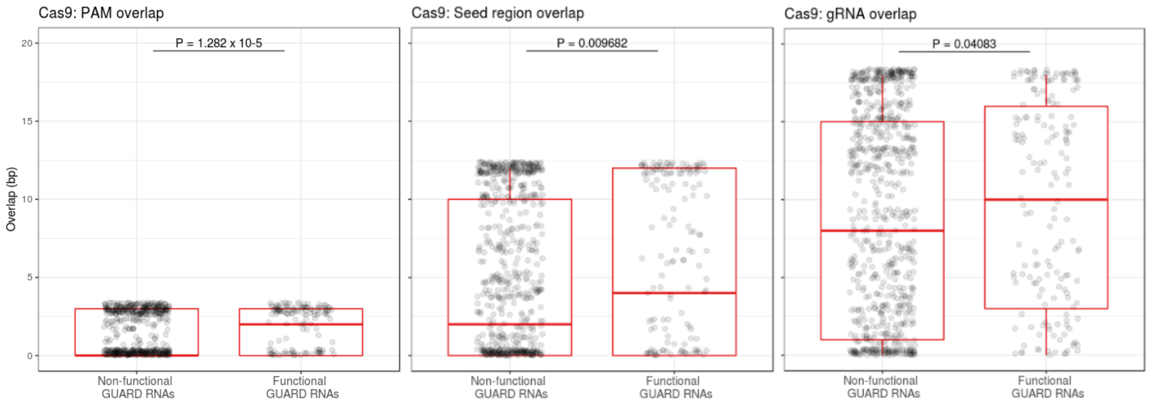

(a) Distribution of functional GUARD RNAs found in the Cas9 screen with increasing GUARD RNA GC-content (%GC).

(b) Functional GUARD RNAs have more overlap with the off-target gRNA binding region. Overlap with the gRNA PAM, seed region including the PAM and entire protospacer sequence including the PAM (bp) was compared for non-functional and functional GUARD RNAs from the Cas9 screen using an unpaired, two-tailed student's *t*-test. Center line, median; box limits, upper and lower quartiles; whiskers, 1.5x interquartile range. Unpaired, two-tailed student's *t*-test.

Supplementary Figure 12

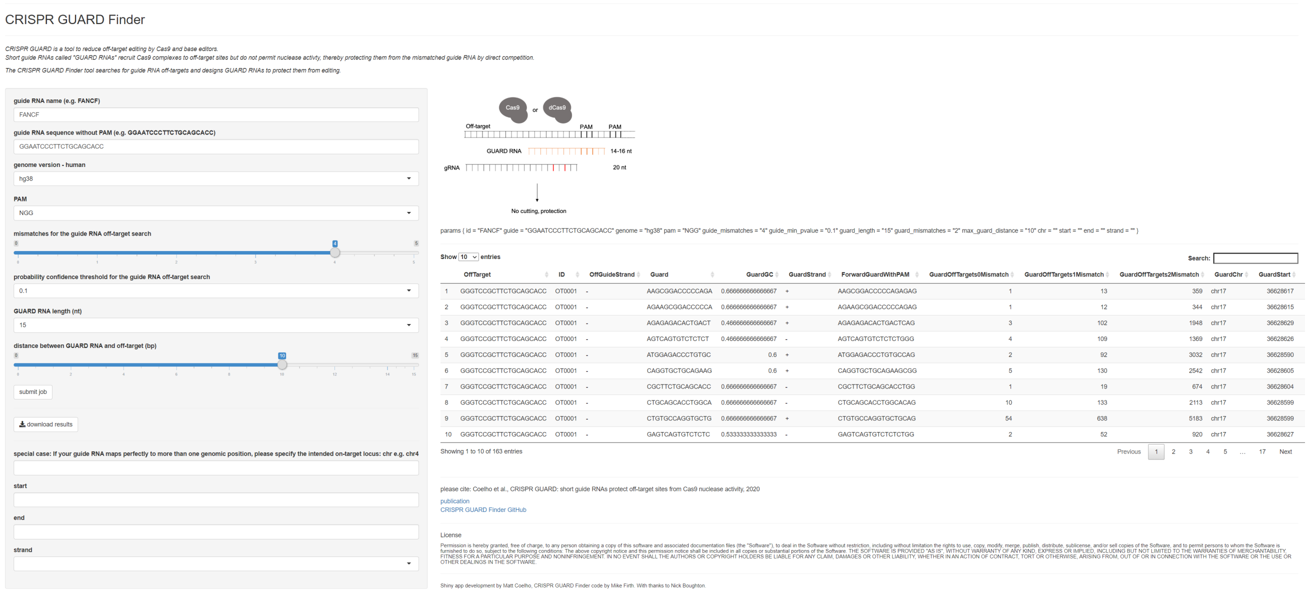

An example workflow of running the “CRISPR GUARD Finder” tool. The user defines parameters such as PAM, guide RNA sequence, search genome, off-target probability, mismatches and GUARD RNA length. The output contains predicted off-targets, possible GUARD RNAs and their relevant sequence features.

Supplementary Table 1

No detectable Cas9-induced indels caused by CRISPR GUARD

Sample	Amplicon	Total reads	% Reads with indel
EMX1 Untransfected	*EMX1*	812998	0.00
EMX1 gRNA only - Positive control	*EMX1*	983231	81.98
CAVIN4 Untransfected	*CAVIN4*	512079	0.34
CAVIN4 14 nt GUARD	*CAVIN4*	1007520	0.33
CAVIN4 15 nt GUARD	*CAVIN4*	1076240	0.35
HCN1 Untransfected	*HCN1*	1674461	0.00
HCN1 14 nt GUARD	*HCN1*	1465594	0.00
MYC prox. Untransfected	*Myc* prox.	1718187	0.10
MYC prox. 14 nt GUARD	*Myc* prox.	1589391	0.10
TENT4A Untransfected	*TENT4A*	1293639	0.00
TENT4A 14 nt GUARD	*TENT4A*	1334905	0.00

Indel rates from NGS of amplicons from Cas9-expressing HEK293 cells transfected with *EMX1* on-target gRNA (full-length, 20-nt guide RNA as a control), or an array of different functional GUARD RNAs at 125 nM.

Supplementary Table 2

List of guide RNAs and GUARD RNAs

RNA molecules	Sequence
EMX1 gRNA	GAGTCCGAGCAGAAGAAGAA**GGG**
EMX1 on-target GUARD	CGAGCAGAAGAAGAA**GGG**
EMX1 OT1 HCN1 GUARD	AAGGCATGGAGTAA**AGG**
EMX1 OT2 MFAP1 GUARD	TAAGCAGAAGAAGAA**GAG**
EMX1 OT3 MYC proximal GUARD	GACTCAGGTCATGC**TGG**
VEGFA gRNA	GACCCCCTCCACCCCGCCTC**CGG**
VEGFA on-target GUARD	CCCGCGCCCGGAGG**CGG**
VEGFA OT1 TENT4A GUARD	CCTCCGGGCAAGTG**AGG**
VEGFA OT2 HDLBP GUARD 1	TTTATTCTTTCCTG**AGG**
VEGFA OT2 HDLBP GUARD 2	TTCTTTCCTGAGGCG**GGG**
VEGFA OT3 CAVIN4 proximal GUARD	TCAGGACGTCCAAC**AGG**
VEGFA OT3 CAVIN4 competitive 15 nt GUARD	CCCCCACCCCGCCTC**CGG**
VEGFA OT3 CAVIN4 proximal 15 nt GUARD	CTCAGGACGTCCAAC**AGG**
VEGFA OT3 CAVIN4 proximal with spacer GUARD	AATTACTCAGGACGTCCAAC**AGG**
HBB gRNA	CTTGCCCCACAGGGCAGTAA**CGG**
FANCF gRNA	GGAATCCCTTCTGCAGCACC**TGG**
HEKsite1 gRNA	GGGAAAGACCCAGCATCCGT**GGG**
Non-targeting gRNA (NT) IDT	GCCCCGCCGCCCTCCCCTCC
Non-targeting (NT) GUARD 1 screen (Avana NO_CURRENT_1)	AAGAGGAATAGTAGC
Non-targeting (NT) GUARD 2 screen (Avana NO_CURRENT_2)	GAAACGTGACTAAAG
Non-targeting (NT) GUARD 3 screen (Avana NO_CURRENT_3)	TTAGGCACGGTTACT

PAMs are indicated in bold.

Supplementary Table 3

List of primers

Primer name	Sequence
qPCR EMX1 For.	5′-TCCAGAACCGGAGGACAAAG-3′
qPCR EMX1 Rev.	5′-CACCGGTTGATGTGATGGGA-3′
qPCR EMX1 OT1 HCN1 For.	5′-TTCTGAGGGCTGCTACCTGT-3′
qPCR EMX1 OT1 HCN1 Rev.	5′-GAGTGGCCAGAGTCCAGCTTGG-3′
qPCR EMX1 OT2 MFAP1 For.	5′-CACGGCCTTTGCAAATAGAG-3′
qPCR EMX1 OT2 MFAP1 Rev.	5′-GGCTTTCACAAGGATGCAGT-3′
qPCR EMX1 OT3 MYC prox. For.	5′-CCAGACTCAGTAAAGCCTGGA-3′
qPCR EMX1 OT3 MYC prox. Rev.	5′-TGGCCCCAGTCTCTCTTCTA-3′
qPCR VEGFA For.	5′-CTGACCAGTCGCGCTGACGG-3′
qPCR VEGFA Rev.	5′-CAGAAGTTGGACGAAAAGTTTCAGTGCG-3′
qPCR VEGFA OT1 TENT4A For.	5′-TCCTACAAGTAACAGTCCAAGAA-3′
qPCR VEGFA OT1 TENT4A Rev.	5′-TTCTGCAACTTAACTTACGTGAAA-3′
qPCR VEGFA OT2 HDLBP For.	5′-ACCAAGCCCATTTGTCCAGG-3′
qPCR VEGFA OT2 HDLBP Rev.	5′-TCCTTCTTTTTGAGCTTTGGGC-3′
qPCR VEGFA OT3 CAVIN4 For.	5′-TCCATACCAGCAGCAGTTCC-3′
qPCR VEGFA OT3 CAVIN4 Rev.	5′-CTCACCTCAGCTCCTGCAC-3′
Amplicon Seq. EMX1 For.	5′-**TCG TCG GCA GCG TCA GAT GTG TAT AAG AGA CAG** TGA GTT TCT CAT CTG TGC CCC-3′
Amplicon Seq. EMX1 Rev.	5′-**GTC TCG TGG GCT CGG AGA TGT GTA TAA GAG ACA G**GT TTG TGG TTG CCC ACC CTA-3′
Amplicon Seq. EMX1 OT1 HCN1 For.	5′-**TCG TCG GCA GCG TCA GAT GTG TAT AAG AGA CAG** TTC TGA GGG CTG CTA CCT GT-3′
Amplicon Seq. EMX1 OT1 HCN1 Rev.	5′-**GTC TCG TGG GCT CGG AGA TGT GTA TAA GAG ACA G**GC CCA ATC ATT GAT GCT TTT-3′
Amplicon Seq. EMX1 OT2 MFAP1 For.	5′-**TCG TCG GCA GCG TCA GAT GTG TAT AAG AGA CAG** CAC GGC CTT TGC AAA TAG AG-3′
Amplicon Seq. EMX1 OT2 MFAP1 Rev.	5′-**GTC TCG TGG GCT CGG AGA TGT GTA TAA GAG ACA G**GG CTT TCA CAA GGA TGC AGT-3′
Amplicon Seq. EMX1 OT3 MYC prox. For.	5′-**TCG TCG GCA GCG TCA GAT GTG TAT AAG AGA CAG** CCA GAC TCA GTA AAG CCT GGA-3′
Amplicon Seq. EMX1 OT3 MYC prox. Rev.	5′-**GTC TCG TGG GCT CGG AGA TGT GTA TAA GAG ACA G**TG GCC CCA GTC TCT CTT CTA-3′
Amplicon Seq. VEGFA For.	5′-**TCG TCG GCA GCG TCA GAT GTG TAT AAG AGA CAG** GGA CAG ACA GAC AGA CAC CG-3′

Amplicon Seq. VEGFA Rev.	5′-**GTC TCG TGG GCT CGG AGA TGT GTA TAA GAG ACA G**GC CCG AGC TAG CAC TTC TC-3′
Amplicon Seq. OT1 VEGFA TENT4A For.	5′-**TCG TCG GCA GCG TCA GAT GTG TAT AAG AGA CAG** TCC TAC AAG TAA CAG TCC AAG AA-3′
Amplicon Seq. OT1 VEGFA TENT4A Rev.	5′-**GTC TCG TGG GCT CGG AGA TGT GTA TAA GAG ACA G**TT CTG CAA CTT AAC TTA CGT GAA A-3′
Amplicon Seq. OT2 VEGFA HDLBP For.	5′-**TCG TCG GCA GCG TCA GAT GTG TAT AAG AGA CAG** ACC AAG CCC ATT TGT CCA GG-3′
Amplicon Seq. OT2 VEGFA HDLBP Rev.	5′-**GTC TCG TGG GCT CGG AGA TGT GTA TAA GAG ACA G**TC CTT CTT TTT GAG CTT TGG GC-3′
Amplicon Seq. OT3 VEGFA CAVIN4 For.	5′-**TCG TCG GCA GCG TCA GAT GTG TAT AAG AGA CAG** TCC ATA CCA GCA GCA GTT CC-3′
Amplicon Seq. OT3 VEGFA CAVIN4 Rev.	5′-**GTC TCG TGG GCT CGG AGA TGT GTA TAA GAG ACA G**CT CAC CTC AGC TCC TGC AC-3′

In bold are adapter sequences used for the addition of next-generation indexing primers.
