# Supplementary material for: CRISPR GUARD protects off-target sites from Cas9 nuclease activity using short guide RNAs
Source: Nat Commun. 2020 Aug 17;11:4132. doi: 10.1038/s41467-020-17952-5 (PMC7431537; doi:10.1038/s41467-020-17952-5)
Supplement: Supplementary file 3 — Reporting Summary [file 41467_2020_17952_MOESM3_ESM.pdf]

nature research

Corresponding author(s): Benjamin Taylor & Matthew Coelho

Last updated by author(s): Mar 18, 2020

Reporting Summary

Nature Research wishes to improve the reproducibility of the work that we publish. This form provides structure for consistency and transparency in reporting. For further information on Nature Research policies, see Authors & Referees and the Editorial Policy Checklist .

Statistics

For all statistical analyses, confirm that the following items are present in the figure legend, table legend, main text, or Methods section.

n/a	Confirmed	
☐	☒	The exact sample size (*n*) for each experimental group/condition, given as a discrete number and unit of measurement
☐	☒	A statement on whether measurements were taken from distinct samples or whether the same sample was measured repeatedly
☐	☒	The statistical test(s) used AND whether they are one- or two-sided<br>*Only common tests should be described solely by name; describe more complex techniques in the Methods section.*
☐	☒	A description of all covariates tested
☐	☒	A description of any assumptions or corrections, such as tests of normality and adjustment for multiple comparisons
☐	☒	A full description of the statistical parameters including central tendency (e.g. means) or other basic estimates (e.g. regression coefficient) AND variation (e.g. standard deviation) or associated estimates of uncertainty (e.g. confidence intervals)
☐	☒	For null hypothesis testing, the test statistic (e.g. *F*, *t*, *r*) with confidence intervals, effect sizes, degrees of freedom and *P* value noted<br>*Give P values as exact values whenever suitable.*
☒	☐	For Bayesian analysis, information on the choice of priors and Markov chain Monte Carlo settings
☒	☐	For hierarchical and complex designs, identification of the appropriate level for tests and full reporting of outcomes
☒	☐	Estimates of effect sizes (e.g. Cohen's *d*, Pearson's *r*), indicating how they were calculated

Our web collection on statistics for biologists contains articles on many of the points above.

Software and code

Policy information about availability of computer code

Data collection	No software was used for data collection
Data analysis	All code for CRISPR GUARD Finder and analyses in this paper including specification of package versions is available from:<br>https://github.com/MatthewACoelho/CRISPRGUARDfinder<br>For amplicon sequencing analysis we used:<br>dpAlign from BioPerl: Bio::Tools::dpAlign (version 1.007001).<br>CRISPR GUARD Finder requires R (version 4.0.0) with optparse (version 1.6.6) and BSgenome (version 1.56.0) packages for each of the genomes required, and nextflow (version 20.01.0).<br>R analysis required "tidyverse" (version 1.3.0).<br>The Shiny app is available online (https://www.sanger.ac.uk/tool/crispr-guard-finder/), but local installation requires further installation of the R packages "shiny" (version 1.4.0.2), "tidyverse" (version 1.3.0), "digest" (version 0.6.25) and "DT" (version. 0.13).

For manuscripts utilizing custom algorithms or software that are central to the research but not yet described in published literature, software must be made available to editors/reviewers. We strongly encourage code deposition in a community repository (e.g. GitHub). See the Nature Research guidelines for submitting code & software for further information.

Data

Policy information about availability of data

All manuscripts must include a data availability statement. This statement should provide the following information, where applicable:

- Accession codes, unique identifiers, or web links for publicly available datasets
- A list of figures that have associated raw data
- A description of any restrictions on data availability

All data generated or analysed during this study are included in this published article, its supplementary information files, and publicly available repositories. Sequencing data is available from the NCBI Sequence Read Archive database, accession SRP252950, BioProject accession PRJNA612602.

October 2018

[https://www.ncbi.nlm.nih.gov/bioproject/PRJNA612602/]
The source data underlying Figures 2, 3, 4 and 6, and Supplementary Figures 2, 3, 4, 5 and 6, are provided as a Source Data file.

Field-specific reporting

Please select the one below that is the best fit for your research. If you are not sure, read the appropriate sections before making your selection.

- [x] Life sciences
- [ ] Behavioural & social sciences
- [ ] Ecological, evolutionary & environmental sciences

For a reference copy of the document with all sections, see nature.com/documents/nr-reporting-summary-flat.pdf

Life sciences study design

All studies must disclose on these points even when the disclosure is negative.

Sample size	No sample size calculations were performed. Presented sample sizes yield statistically significant differences between biological samples.
Data exclusions	No data was excluded from analysis.
Replication	All experiments were replicated by performing independent repeat experiments on different days to ensure reproducibility of our findings. All attempts to replicate results were successful.
Randomization	No randomisation was required as cell cultures were treated and grown identically apart from the experimental perturbation
Blinding	No blinding was required as cell cultures were treated and grown identically apart from the experimental perturbation

Reporting for specific materials, systems and methods

We require information from authors about some types of materials, experimental systems and methods used in many studies. Here, indicate whether each material, system or method listed is relevant to your study. If you are not sure if a list item applies to your research, read the appropriate section before selecting a response.

Materials & experimental systems

n/a	Involved in the study	
x		Antibodies
	x	Eukaryotic cell lines
x		Palaeontology
x		Animals and other organisms
x		Human research participants
x		Clinical data

Methods

n/a	Involved in the study	
x		ChIP-seq
x		Flow cytometry
x		MRI-based neuroimaging

Eukaryotic cell lines

Policy information about cell lines

Cell line source(s)	HEK293 cells were obtained from ATCC
Authentication	STR profiling
Mycoplasma contamination	All cell lines used in this study tested negative for mycoplasma contamination
Commonly misidentified lines (See ICLAC register)	No commonly misidentified cell lines were used in the study
